# Isomeric 3-Pyridinylmethylcoumarins Differ in Erk1/2-Inhibition and Modulation of BV2 Microglia-Mediated Neuroinflammation

**DOI:** 10.3390/molecules30112452

**Published:** 2025-06-03

**Authors:** Rami Schulzki, Matthias Apweiler, Caroline Röttger, Christoph W. Grathwol, Nora Struchtrup, Sophia Abou El Mirate, Claus Normann, Stefan Bräse, Bernd L. Fiebich

**Affiliations:** 1 Neuroimmunology and Neurochemistry Research Group, Department of Psychiatry and Psychotherapy, Medical Center-University of Freiburg, Faculty of Medicine, University of Freiburg, D-79104 Freiburg, Germany; rischulzki@gmail.com (R.S.); matthias.apweiler@uniklinik-freiburg.de (M.A.); 2 Institute of Organic Chemistry (IOC), Karlsruhe Institute of Technology (KIT), Kaiserstraße 12, D-76131 Karlsruhe, Germany; caroline.roettger@kit.edu (C.R.); noramarie@shaw.ca (N.S.); sophia.mirate@kit.edu (S.A.E.M.); braese@kit.edu (S.B.); 3 Institute of Biological and Chemical Systems-Functional Molecular Systems (IBCS-FMS), Karlsruhe Institute of Technology (KIT), Kaiserstraße 12, D-76131 Karlsruhe, Germany; christoph.grathwol@kit.edu; 4 Mechanisms of Depression Research Group, Department of Psychiatry and Psychotherapy, Medical Center-University of Freiburg, Faculty of Medicine, University of Freiburg, D-79104 Freiburg, Germany; claus.normann@uniklinik-freiburg.de

**Keywords:** coumarins, isomeric, Erk1/2, MAPK, microglia, neuroinflammation, cytokines, chemokines, GPR55

## Abstract

Coumarins are known for their multiple biological effects and have been established as anti-coagulative drugs for years. Furthermore, some coumarins can promote anti-inflammatory effects via the GPR55 receptor, and dual target coumarins have been synthesized. Anti-inflammatory drugs might be beneficial in the treatment of neuropsychiatric disorders, as the inflammatory hypothesis suggests. For the current study, we compared isomeric 3-pyridinylmethylcoumarins with altered N-atom position regarding their effects on cytokine and chemokine synthesis and expression in LPS-stimulated BV2 microglial cells. The 3-pyridin-4-yl-methylcoumarin showed the most potent anti-inflammatory effects, followed by the 3-pyridin-2-ylmethylcoumarin analog. The observed effects might be mediated by an inhibition of ERK phosphorylation.

## 1. Introduction

Coumarins have been known for their multiple biological effects for years. For example, the anti-coagulative effect of warfarin by the inhibition of the γ-carboxylation in the synthesis of the vitamin K-dependent clotting factors [1] established coumarins as valuable pharmaceutics. Depending on the chemical residues added to the coumarin scaffold, anti-microbial, anti-proliferative, anti-oxidative, and anti-inflammatory effects have been described in addition to its already mentioned anti-coagulative effects [2]. The coumarin scaffold consists of a benzene ring fused to a α-pyrone, provoking biological activity through hydrogen bonding and hydrophobic interactions with target structures [2]. In an investigation on the selectivity of various coumarins towards the cannabinoid receptors and G-protein-coupled receptor (GPR) 55, substituents on C-5 of the coumarin scaffold were found to be essential for bioactivity. Instead, substituents on C-7 had an influence on the cannabinoid receptors (CB) affinity only and additional substituents on C-8 lead to an antagonistic activity towards GPR55 [3]. In one of our previous works, we showed that coumarins with residues on C-3, -5, and -8 have anti-inflammatory and anti-oxidative effects [4,5,6,7] in primary microglial cells, most likely due to an inverse agonism on GPR55. Another coumarin derivative, KIT 10, with substituents on C-3, a methoxy group at C-5, and a 1-butylcyclohexyl residue at C-7, equally exerted anti-inflammatory effects probably dependent on GPR55, highlighting the relevance of C-3 and C-5 substitution compared to C-8 [7]. Since specific substitution patterns on the coumarin scaffold allow us to adjust the selectivity and affinity towards different target receptors, further research into this pharmacophore has been undertaken [8]. For example, a coumarin–dithiocarbamate hybrid showed inhibitory effects on both acetylcholinesterase (AChE) and monoaminoxidase B (MAO B) and reversed scopolamine-induced memory deficits in mice, underlining the potential of this structural class for the treatment of neurodegenerative diseases, such as Alzheimer’s disease (AD) [9].

In recent years, GPR55 has gained importance in the treatment of neuropsychiatric disorders. For example, O-1602, a synthetic GPR55 agonist reversed β amyloid-induced cognitive impairment and release of pro-inflammatory cytokines as well as the production of reactive oxygen species and cell death in mice, suggesting GPR55 as a promising target to treat AD [10]. Similar effects were observed in streptozotocin-induced murine AD models [11]. Furthermore, the role of GPR55 in substance use disorders [12], depressive symptoms [13], suicide [14], and anxiety [15] has been proposed. Therefore, a pharmacological intervention via GPR55 and the activation of associated anti-inflammatory pathways might be a promising and new approach in the treatment of various neuropsychiatric disorders.

Studies support the hypothesis that anti-inflammatory pharmaceutics might be beneficial in the treatment of neuropsychiatric disorders. Elevated interleukin (IL)-6 plasma concentrations were measured in elderly patients with major depression [16]. Another study did not show significant differences in IL-6 levels between patients with depression and healthy controls; however, treatment with selective serotonin reuptake inhibitors (SSRIs) significantly decreased peripheral IL-6 levels in both patients and controls [17]. Another study showed an association between poorer psychopharmaceutical response to SSRIs or selective serotonin and noradrenalin reuptake inhibitors (SNRIs) treatment and high peripheral IL-6 levels [18]. The relevance of inflammatory processes in AD was shown in post-mortem brain analyses of patients showing IL-6 immunoreactivity in β amyloid plaques in AD but not in brain plaques of age-matched controls [19]. Interestingly, plasma IL-6 levels were negatively correlated with hippocampal gray matter density and scoring in the mini-mental state examination (MMSE) [19]. Targeting those inflammatory processes has gained interest in the scientific community in recent years and lead to the development of new compounds and conduction of clinical trials, underlining the relevance of anti-inflammatory drug research in psychiatric disorders. For example, the inhibition of intracellular cortisone reactivation via 11β-hydroxysteroid dehydrogenase (11β-HSD1) is discussed as another promising way to ameliorate neuroinflammation in psychiatric disorders, especially AD. In some studies, reduction in cognitive decline as well as reduced inflammatory responses have been observed under treatment with 11β-HSD1 inhibitors in preclinical studies, however, the results in the subsequent clinical trials were inconsistent over different studies [20]. Excessive cortisol levels lead to an internalization of glucocorticoid-receptors responsible for the anti-inflammatory actions and—in contrast to normal levels—do not inhibit pro-inflammatory cytokine release for that reason [21]. 11β-HSD activity was associated with the severity of depressive symptoms measured using the Hamilton Depression Rating Scale (HDRS) in women but not men [22]. In AD, 11β-HSD1 knockout or inhibition prevented cognitive impairments and memory deficits, but effects might only be relevant in age-related cognitive decline [23]. However, the effects in clinical studies are not consistent and some 11β-HSD1 inhibitors failed to achieve the primary endpoints, though higher dosages might be necessary [23,24].

Targets of the anti-inflammatory treatment are the central nervous system’s residual immune cells, the microglial cells. Microglia are responsible for the pro- and anti-inflammatory homeostasis of the CNS under physiological conditions [25]. However, in case of pathogen exposition, damage or auto-immunological reactions, microglia modulate a pro-inflammatory response as well as production of reactive oxygen species in order to attract and differentiate further immune cells to restrict the consequences of the exposition. The aim of this cellular response is to remove pathogens, dead cells, and to restore the brain’s homeostasis [25]. As highlighted before, involvement of cytokines, such as IL-6, is discussed in the genesis and progression of neuropsychiatric disorders. However, also chemokines, such as C-C motif chemokine ligand (CCL) 2, also referred to as “monocyte chemoattractant protein 1” (MIP-1), were, for example, elevated in patients with mild or moderate major depressive disorder (MDD) in comparison to healthy controls and normalized after an internet-based cognitive-behavioral intervention [26]. In patients with Parkinson’s Disease (PD), peripheral concentrations of the chemokine C-X-C motif chemokine ligand (CXCL) 10, also known as interferon gamma induced protein 10 (IP-10), were positively correlated with cognitive deficits, disease’s progression but not depressive symptoms [27]. Furthermore, higher CXCL10 levels were observed in patients with major depressive disorders compared to healthy controls and were even significantly enhanced after 8 weeks treatment with the SSRI fluoxetine in one study [28]. Another study supported the finding of higher peripheral CXCL10 concentrations in patients with MDD. In contrast to the previously mentioned study, patients who responded to an antidepressive treatment (fluoxetine or desipramine), showed a significant decrease in CXCL10 levels instead [29]. Therefore, the role of cytokines and chemokines still needs further investigations to clarify their potential for diagnostic and therapeutic applications in neuropsychiatric disorders. While rare cases of auto-antibody-mediated organic psychiatric disorders have already resulted in new therapeutic options [30], no inflammatory biomarkers have been established so far [31].

Nevertheless, some patients with psychiatric disorders seem to benefit from anti-inflammatory treatment. A systematic review suggests an improvement in depressive symptoms after treatment with different anti-inflammatory substances as monotherapy or add-on therapy to antidepressants [32]. Another review reported benefits on depressive symptoms after treatment with non-selective cyclooxygenase (COX) inhibitors as well as COX-2 selective pharmaceutics [33]. Patients with an inflammatory comorbidity and depression, in particular, might profit by including an anti-inflammatory drug in the therapeutic regime [33].

**Figure 1:**
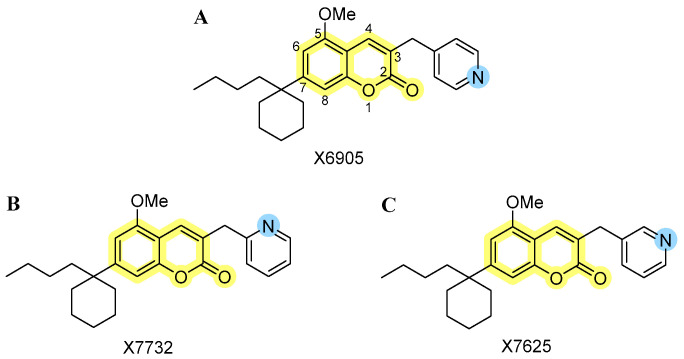
Chemical structures of the investigated isomeric 3-pyridinylmethylcoumarins: (**A**) 3-pyridin-**4**-ylmethylcoumarin (X6905), (**B**) 3-pyridin-**2**-ylmethylcoumarin (X7732), and (**C**) 3-pyridin-**3**-ylmethylcoumarin (X7625). The coumarin core is highlighted in yellow, along with the varying *para*-, *ortho*- and *meta*-position of the nitrogen atom (blue) within the pyridinyl moiety.

Since coumarins are known for their anti-inflammatory effects and might act on multiple molecular pathways and targets via different receptors, the current study focuses on anti-inflammatory effects of three novel isomeric 3-pyridinylmethylcoumarins. We were especially interested in the biological significance of small structural changes in the pyridine subunit. The effects of X6905 (Figure 1A, represented as light blue color scheme in result figures), X7732 (Figure 1B, represented as blue color scheme in result figures), and X7625 (Figure 1C, represented as dark blue color scheme in result figures) on cytokine and chemokine release were evaluated in lipopolysaccharide (LPS)-stimulated BV2 microglial cells. Furthermore, pathways that were possibly involved were examined focusing on mitogen-activated protein kinases (MAPKs).

## 2. Results

### 2.1. Effects of X6905, X7732, and X7625 on Cell Viability (ATP -Assay)

**Figure 2:**
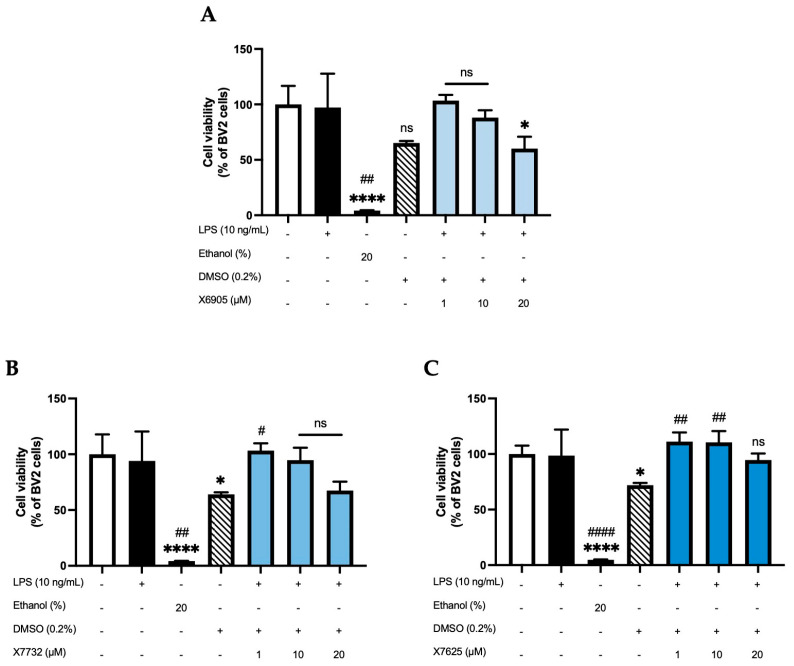
Effects of X6905-light blue bars (**A**), X7732—blue bars (**B**), and X7625—dark blue bars (**C**) on cell viability of LPS-stimulated BV2 cells after 24 h of stimulation. Cell viability was assessed using an ATP assay. Values are shown as the mean of 3 (ethanol—dark bar, DMSO—striped bar, coumarins) or 6 (control—white bar and LPS group—black bar) individual measurements ± SDs. A one-way ANOVA was performed for statistical analysis using the untreated cells (white bar) or the 0.2% DMSO-group (striped bar) as reference for Dunett’s post-hoc test. Asterisks compare to the control-group with * *p* < 0.05 and **** *p* < 0.0001, while hashtags are used for 0.2% DMSO-reference with # *p* < 0.05; ## *p* < 0.01 and #### *p* < 0.0001. Non-significant differences are labeled as “ns”.

An ATP assay was performed to assess whether the compounds X6905, X7732, and X7625 (1, 10, 20 µM) have an influence on cell viability in LPS-stimulated BV2 cells (Figure 2). Cell viability was not affected by the stimulation with LPS, while 20% ethanol significantly induced cell death. DMSO was used as solvent for all compounds. Accordingly, a 0.2% DMSO group was included as a vehicle control. DMSO decreased cell viability to approximately 70% of untreated BV2 cells. X6905 and X7732 similarly reduced cell viability to around 70% at 20 µM, while X7625 had a rather promoting effect at lower concentrations. The only significant reduction in cell viability was observed for X6905 at 20 µM. To exclude a DMSO-mediated effect, a second one-way ANOVA was performed using 0.2% DMSO as reference (indicated with #), which showed no significant reduction in cell viability in BV2 cells for any compound.

### 2.2. Effects of X6905, X7732, and X7625 on LPS-Induced Cytokine Release

Next, a potential compound-dependent modulation of LPS-induced cytokine release was investigated by measuring cytokine concentrations of IL-6, CCL2, CXCL2, CXCL10, and TNF-α in cell supernatant after 24 h of simulation with LPS in the absence or presence of the respective coumarin derivative at different concentrations (0.1, 1, 5, 10, 20 µM).

#### 2.2.1. IL-6 Release

**Figure 3:**
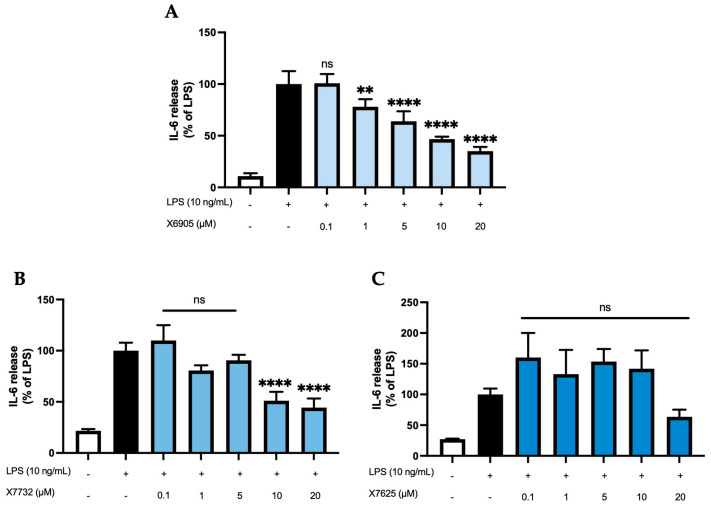
Effects of X6905—light blue bars (**A**), X7732—blue bars (**B**), and X7625—dark blue bars (**C**) on LPS-induced release of IL-6 in BV2 cells. IL-6 concentrations were measured in the supernatant using ELISA. The values were normalized to the LPS response (black bar) and are shown as the mean of three to six (*n* = six for the control and LPS groups in A and *n* = three for all other groups) individual measurements ± SDs. For statistical analysis, one-way ANOVA with Dunett’s post-hoc test was performed using the LPS group as reference with ** *p* < 0.01 and **** *p* < 0.0001. Non-significant differences are labeled as “ns”.

Figure 3 shows the effects of X6905 (A), X7732 (B), and X7625 (C) on LPS-induced IL-6 release. IL-6 release of BV2 cells was strongly induced after 24 h of LPS stimulation, being 9- (A), 4.5- (B), or 3.5- (C) times higher compared to the control group. X6905 showed a significant and dose-dependent reduction in IL-6 to 35% of LPS at 20 µM. A steady reduction in IL-6 was visible, starting at 1 µM, where IL-6 was already reduced to 78% of the LPS response. In higher concentrations, highly significant results were obtained. X7732 had a similar but slightly weaker effect compared to X6905. At 20 µM, X7732 reduced LPS-induced IL-6 release by 56%. X7625 did not show a dose-dependent reduction in LPS-induced IL-6 release. Only at 20 µM, X7625 reduced the release of IL-6 to 63% of the LPS response. At lower concentrations (0.1 to 1 µM), a slight, though statistically not significant, increase in IL-6 release, with 33% and 55% above the LPS group, was observed.

#### 2.2.2. CCL2 Release

**Figure 4:**
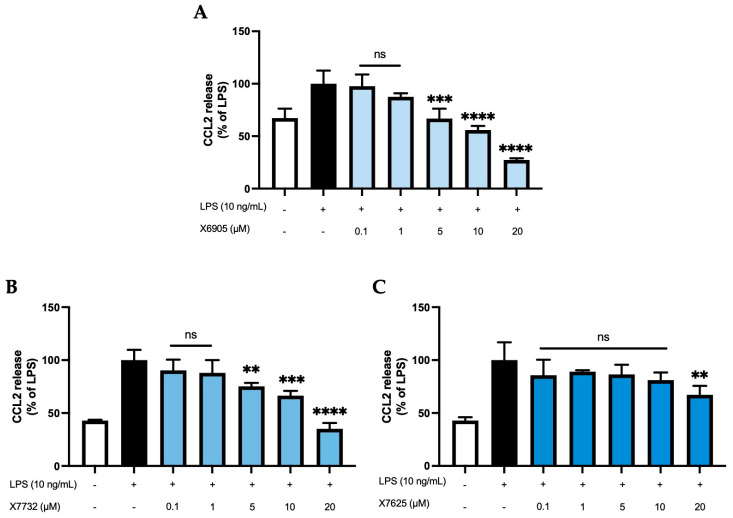
Effects of X6905—light blue bars (**A**), X7732—blue bars (**B**), and X7625—dark blue bars (**C**) on LPS-induced release of CCL2 in BV2 cells. CCL2 concentrations were measured in the supernatant using ELISA. The values were normalized to LPS (black bar) and are shown as the mean of three to six (*n* = six for the control and LPS groups in A and *n* = three for all other groups) individual measurements ± SDs. For statistical analysis, one-way ANOVA with Dunett’s post-hoc test was performed using the LPS group as reference with ** *p* < 0.01; *** *p* < 0.001 and **** *p* < 0.0001. Non-significant differences are labeled as “ns”.

CCL2 release of BV2 cells (Figure 4) was studied after 24 h of treatment with the coumarins X6905 (A), X7732 (B), and X7625 (C) in the presence of LPS. LPS increased CCL2 release by 33% (A) or 58% (B and C) of LPS compared to untreated BV2 cells. X6905 and X7732 showed comparable inhibitory effects: Both compounds showed a dose-dependent and significant reduction in LPS-mediated CCL2 release. However, X6905 lowered CCL2 stronger than the corresponding concentrations of X7732. Both exhibited a dose-dependent reduction in CCL2 release between 0.1 µM and 10 µM, followed by a marked drop at 20 µM, reaching 27% of the LPS response for X6905 and 35% for X7732. In contrast, X7625 had no significant effect on LPS-induced CCL2 release at concentrations between 0.1 and 10 µM, with comparably high levels of CCL2 observed. At 20 µM, however, X7625 significantly decreased LPS-stimulated CCL2 release to 70% of the LPS response.

#### 2.2.3. CXCL2 and CXCL10 Release

**Figure 5:**
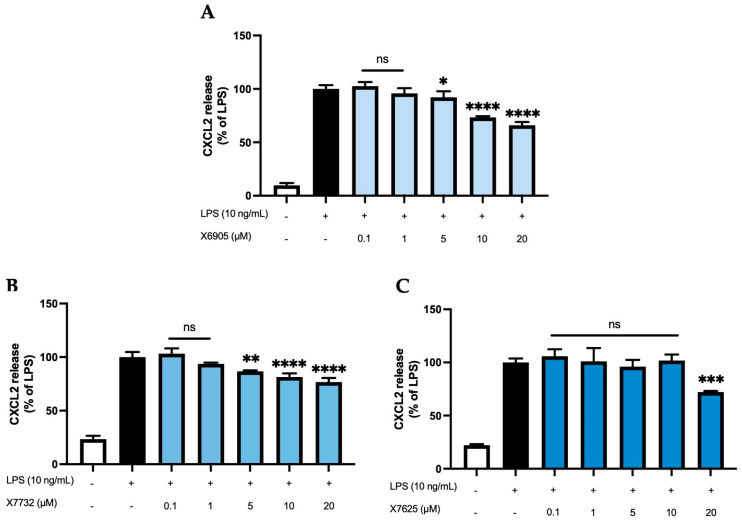
Effects of X6905 – light blue bars (**A**), X7732 – blue bars (**B**), and X7625 – dark blue bars (**C**) on LPS-induced release of CXCL2 in BV2 cells. CXCL2 concentrations were measured in the supernatant using ELISA. The values were normalized to LPS (black bar) and are shown as the mean of three to six (*n* = six for the control and LPS groups in A and *n* = three for all other groups) individual measurements ± SDs. For statistical analysis one-way ANOVA with Dunett’s post-hoc test was performed using the LPS group as reference with * *p* < 0.05; ** *p* < 0.01; *** *p* < 0.001 and **** *p* < 0.0001. Non-significant differences are labeled as “ns”.

**Figure 6:**
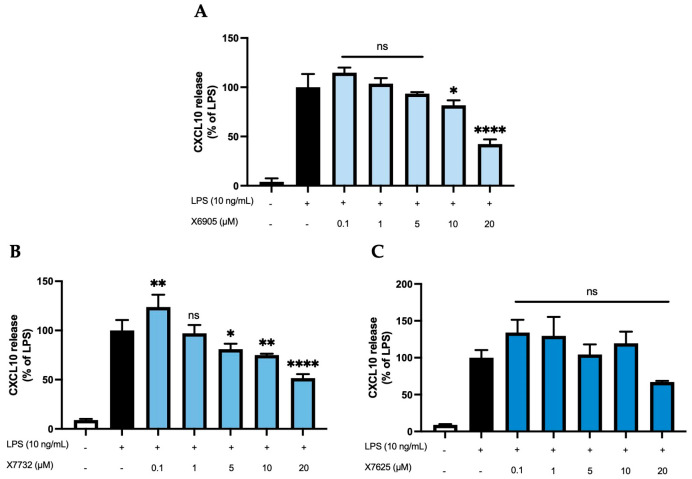
Effects of X6905 – light blue bars (**A**), X7732 – blue bars (**B**), and X7625 – dark blue bars (**C**) on LPS-induced release of CXCL10 in BV2 cells. CXCL10 concentrations were measured in the supernatant using ELISA. The values were normalized to LPS (black bar) and are shown as the mean of three to six (*n* = six for the control and LPS groups in A and *n* = three for all other groups) individual measurements ± SDs. For statistical analysis one-way ANOVA with Dunett’s post-hoc test was performed using the LPS group as reference with * *p* < 0.05; ** *p* < 0.01 and **** *p* < 0.0001. Non-significant differences are labeled as “ns”.

We further studied the effects of X6905 (A), X7732 (B), and X7625 (C) on LPS-induced synthesis of CXCL2 and CXCL10, another group of chemokines in BV2 cells (Figure 5 and Figure 6). After 24 h of stimulation, LPS induced a 10-time (A) and 4-time (B,C) increase in released CXCL2 compared to unstimulated cells. For CXCL10, LPS showed a 25- (A) and 11-times (B,C) induction in comparison to untreated cells. Both X6905 and X7732 exhibited dose-dependent and significant inhibitory effects on the LPS-mediated secretion of CXCL2 and CXCL10. The effect on CXCL10 release was slightly stronger than for CXCL2, although initially an increased CXCL10 level was detected at the lowest concentration of 0.1 µM X7732.

At the highest concentration of 20 µM, CXCL2 release decreased to 66% of the LPS-stimulated cytokine levels for X6905 and to 76% for X7732, respectively. In the case of CXCL10, X6905 decreased the LPS-stimulated response to 42% while X7732 reached 51% of LPS at 20 µM. X7625 showed no dose-dependent effects in the range of 0.1 to 20 µM. Interestingly, it was observed that X7625 rather enhanced LPS-stimulated CXCL10 release in concentrations between 0.1 and 10 µM. Nevertheless, at 20 µM, X7625 decreased the CXCL2 and CXCL10 responses to about 72% and 67% of the LPS-stimulated cells.

#### 2.2.4. TNF-α Release

**Figure 7:**
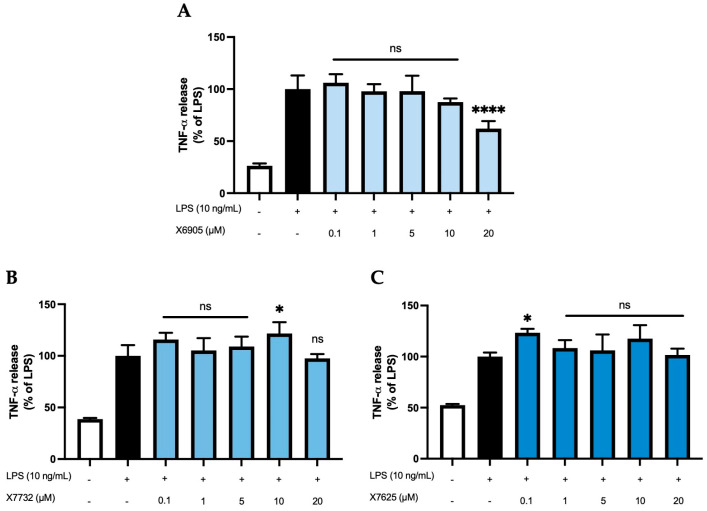
Effects of X6905 – light blue bars (**A**), X7732 – blue bars (**B**), and X7625 – dark blue bars (**C**) on LPS-induced release of TNF-α in BV2 cells after 24 h treatment. TNF-α concentrations were measured in the supernatant using ELISA. The values were normalized to LPS (black bar) and are shown as the mean of three to six (*n* = six for the control and LPS groups in A and *n* = three for all other groups) individual measurements ± SDs. For statistical analysis, one-way ANOVA with Dunett’s post-hoc test was performed using the LPS group as reference with * p < 0.05 and **** *p* < 0.0001. Non-significant differences are labeled as “ns”.

We next studied the effects of X6905 (A), X7732 (B), and X7625 (C) on LPS-induced synthesis of TNF-α, a key inflammatory cytokine involved in neuroinflammation (Figure 7). LPS stimulation led to a four- (A) and two-fold (B,C) increase in TNF-α secretion. X6905 exhibited a modest, dose-dependent decrease in TNF-α levels compared to LPS stimulation. Between 0.1 and 10 µM, a gradual decrease to 87% of the LPS-stimulated response was observed. At 20 µM, the effect of X6905 reached statistical significance with 62% of the LPS-stimulated level.

In contrast, neither X7732 nor X7625 affected LPS-induced TNF-α levels. However, some significant effects were observed: X7732 significantly enhanced LPS-mediated TNF-α release at 10 µM, and X7625 showed a similar effect at 0.1 µM.

### 2.3. Effects of X6905 on LPS-Induced Cytokine Expression

**Figure 8:**
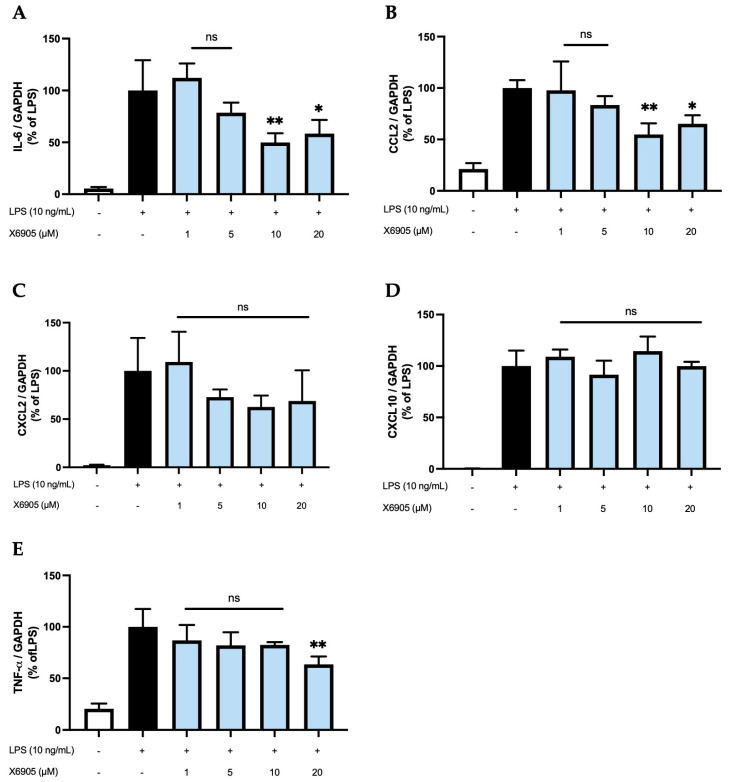
Effects of X6905 (light blue bars) on LPS-induced gene expression of IL-6 (**A**), CCL2 (**B**), CXCL2 (**C**), CXCL10 (**D**), and TNF-α (**E**). Gene expression was quantified using qPCR. The quantity of copies was normalized to consecutively expressed GAPDH. The values were normalized to LPS (black bar) and are shown as the mean of three individual values ± SDs. For statistical analysis, one-way ANOVA with Dunett’s post-hoc test was performed using the LPS group (black bar) as reference with * *p* < 0.05 and ** *p* < 0.01. Non-significant differences are labeled as “ns”.

To analyze whether the effects observed on the protein levels are also detectable on the transcriptional level, we stimulated BV2 cells with different doses of X6905 (1, 5, 10, and 20 µM) in presence of LPS and investigated the expression of IL-6 (A), CCL2 (B), CXCL2 (C), CXCL10 (D), and TNF-α (E) using qPCR (Figure 8). For all cytokines, a considerable induction of expression was observed after LPS stimulation.

IL-6, CCL2, and TNF-α were the only cytokines/chemokines significantly affected by X6905. Treatment with 1, 5, and 10 µM of X6905 led to a gradual decrease on LPS-induced IL-6 expression with a significant reduction of 50% of LPS-stimulated cells at 10 µM. At a concentration of 20 µM, LPS-induced IL-6 expression was significantly reduced to 58%. The influence of X6905 on LPS-mediated CCL2 expression showed a comparable profile: Between 1 and 10 µM, a gradual, dose-dependent decrease in CCL2 expression was observed, reaching 54% of the LPS-stimulated CCL2 expression. At 20 µM, however, a reduction to only 65% compared to LPS was found. X6905 had only weak effects on LPS-induced TNF-α expression, reducing mRNA levels to 82% of LPS-stimulated levels at concentrations of 1, 5, and 10 µM. A significant effect was observed at 20 µM, where the transcription of TNF-α was decreased to 63% of LPS-stimulated levels. LPS-stimulated CXCL10 expression was not affected by X6905.

### 2.4. Effects of X6905, X7732, and X7625 on LPS-Mediated Phosphorylation of Erk1/2

**Figure 9:**
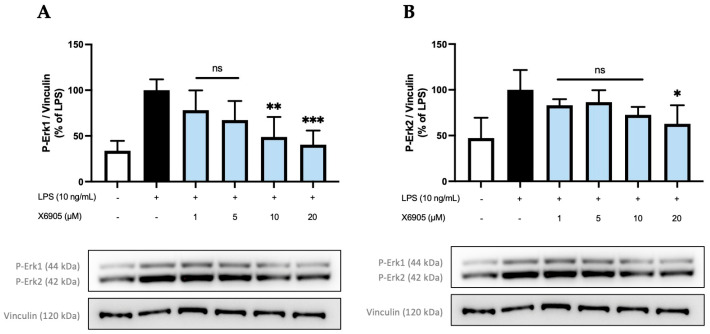
Effects of X6905 (light blue bars) on phospho-Erk1 (**A**) and phospho-Erk2 (**B**), Vinculin was used for reference for equal gel loading. The cells were stimulated with X6905 for 30 min before LPS was added for another 30 min. The values were normalized to LPS (black bar) and are shown as the mean of three–four individual Western blot measurements ± SDs. One-way ANOVA with Dunett’s post-hoc test was performed using the LPS group as reference with * *p* < 0.05; ** *p* < 0.01; *** *p* < 0.001. Non-significant differences are labeled as “ns”.

**Figure 10:**
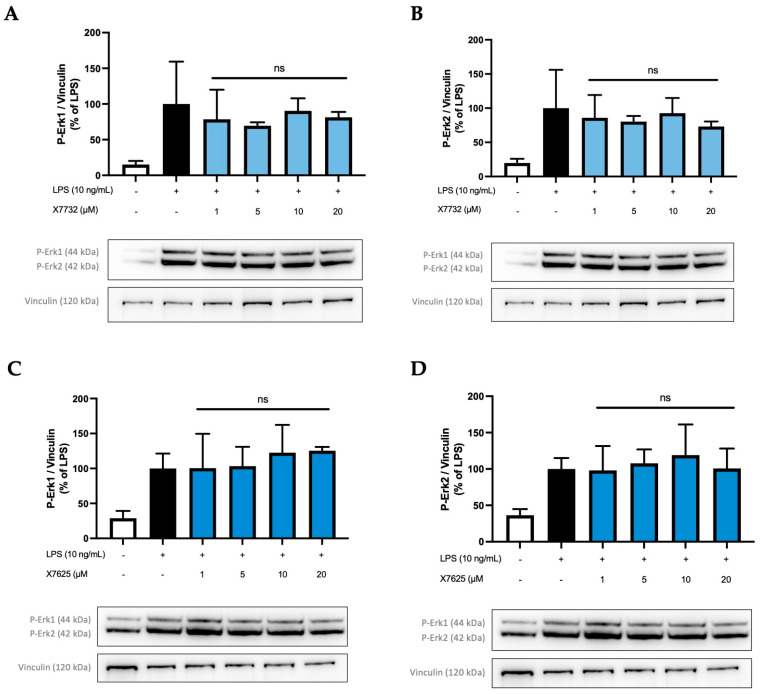
Effects of X7732 (blue bars) and X7625 (dark blue bars) on phosphorylation of Erk1 (**A**,**C**) and Erk2 (**B**,**D**). Vinculin is used for reference for equal gel loading. The cells were stimulated with the compounds for 30 min, before LPS was added for another 30 min. The values were normalized to the LPS value (black bar) and are shown as the mean of three individual Western blot measurements ± SDs. One-way ANOVA with Dunett’s post-hoc test was performed using the LPS group (black bar) which showed no significant (ns) effects.

After investigating the anti-inflammatory potential of X6905, X7732, and X7625, further research was carried out to reveal a possible inhibition of intracellular signaling pathways and kinases such as MAP-Kinases (MAPK) and others. X6905 affected LPS-induced phosphorylation of Erk1/2 (Figure 9), which triggered further investigations of Erk1/2 in response to a treatment with X7732 and X7625 (Figure 10).

The effect of X6905 on phospho-Erk1 (A) and phospho-Erk2 (B) are shown separately. After LPS stimulation, a three- (A) and two-fold (B) increase in phosphorylated Erk1 (A) and Erk2 (B) was detected. The levels of both phosphorylated proteins were significantly decreased by X6905, particularly at higher concentrations. Interestingly, X6905 had differential effects on Erk1 and Erk2 inhibition: A much stronger effect was observed for Erk1, where X6905 decreased phospho-Erk1 levels dose-dependently and significantly. At 1 µM, phospho-Erk1 was already reduced by 22% of the LPS-stimulated group and was further decreased at 20 µM X6905 to almost the level of the unstimulated control to 40% of the LPS-stimulated group. For phospho-Erk2, the effect of X6905 was less pronounced but still significant at a concentration of 20 µM, showing a reduction to 60% of the LPS-stimulated protein level.

To investigate if a similar effect on Erk1 phosphorylation is also caused by the other compounds X7732 and X7625, further investigations were carried out (Figure 10). Phospho-Erk1 (A,C) and phospho-Erk2 (B,D) were again analyzed individually. In all experiments, phosphorylation of Erk1 and Erk2 was induced by LPS-stimulation.

Interestingly, X7732 also exerted a mild inhibitory effect on phospho-Erk1. A decrease was observed at 1 µM and 5 µM, reaching 78% and 70% of LPS-stimulated levels, respectively. At 10 and 20 µM, the levels of phospho-Erk1 increased but remained below the LPS-stimulated levels. For phospho-Erk2, a similar effect was observed, namely a rather weak dose-dependent reduction in phospho-Erk2 to 73% of LPS-stimulated levels. However, the LPS control group showed relatively high standard deviations for phospho-Erk1 and phospho-Erk2. Contrarily, X7625 was observed to increase phospho-Erk1 levels to approximately 120% of the LPS control at 10 and 20 µM, while not affecting phospho-Erk2 levels.

### 2.5. Effects of X6905 on Other Kinases and Signaling Proteins

**Figure 11:**
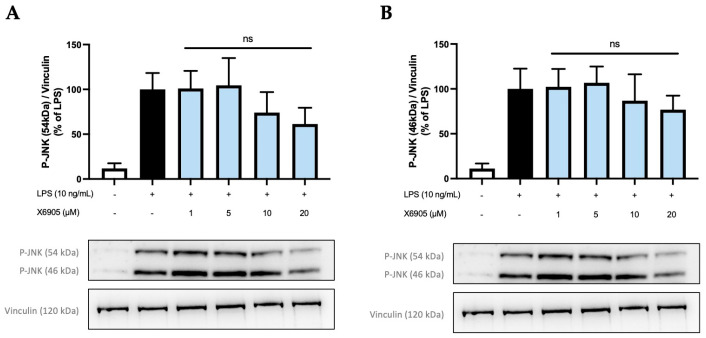
Effects of X6905 (light blue bars) on the phosphorylated subunits of JNK: phospho-JNK (46 kDa) (**A**) and phospho-JNK (54 kDa) (**B**). Vinculin was used for reference for equal gel loading. The cells were stimulated with X6905 for 30 min, before LPS was added for another 30 min. The values were normalized to LPS (black bars) and are shown as the mean of three–four individual Western blot measurements ± SDs. One-way ANOVA with Dunett’s post-hoc test was performed using the LPS group as reference that showed no significant effects (ns).

In addition to Erk1/2, further kinases and signal-transducing proteins were investigated for the potential inhibitory effects of X6905. PKC, p38 MAPK, and IkBα were not modulated after X6905 treatment (Supplementary Materials). Phosphorylation of SAPK/JNK was inhibited by X6905 to a small extent, but no statistical significance was observed (Figure 11). JNK consists of two subunits 46 kDa (A) and 54 kDa (B), which were both extensively phosphorylated after LPS-treatment. X6905 caused a dose-dependent reduction to 76% of the LPS-stimulated levels for the 46 kDa subunit. Interestingly, LPS-induced phosphorylation of the 54 kDa subunit was inhibited by X6905: While X6905 did not affect this subunit at 1 and 5 µM, a dose-dependent decrease to 61% was visible after stimulation at 20 µM.

## 3. Discussion

In this study, we investigated the anti-inflammatory effects of three novel isomeric 3-pyridinylmethylcoumarin derivatives in murine BV2 microglial cells. The aim was to evaluate the biological effects of the different orientations of the pyridine moiety. Only X6905, slightly but significantly, reduced cell viability in the highest concentration of 20 µM. X7732 showed a trend toward reduced cell viability at the highest concentration, although this effect did not reach statistical significance. X6905 strongly inhibited LPS-induced IL-6 release and expression as well as CCL2 release, probably mediated via the Erk1/2 pathway. Furthermore, a significant reduction in CXCL2 and CXCL10 release was observed for X6905. X7732 exhibited comparable effects to X6905 with a slightly reduced biological potency and increased TNF-α release at a concentration of 10 µM, and with an enhancement of CXCL10 release at 0.1 µM. X7625 enhanced TNF-α release at concentrations of 0.1 µM and significantly reduced CXCL10 release at 20 µM; however, the effects were less concentration-dependent. Overall, these results support the anti-inflammatory potential of coumarin derivatives and underline the enormous relevance of small chemical alterations in modulating biological efficacy.

The applied ATP assay is based on a luciferase reaction, allowing the measurement of cellular ATP levels by luminescence after cell lysis. It is known for its high sensitivity and robustness against artifacts compared to other cell viability assays [34]. However, intracellular ATP levels may depend on the cells’ metabolic state [35,36]; therefore, treatment with compounds may influence intracellular ATP levels. Treatment with 0.2% of DMSO, which was used as solvent of the tested coumarin derivatives, reduced cell viability by approximately 30%. To address the cytotoxic effects of DMSO, 0.2% of DMSO was included in all controls (untreated cells and LPS control) in the following experiments. However, the cytotoxic effects of the tested compounds should be further investigated in future studies, including more complex tissue models or in vivo experiments and the possible use of alternative solvents. Some novel aprotic polar solvents, such as zwitterionic liquid [37], might be less cytotoxic than DMSO with comparable solubility of the coumarins and should be compared to DMSO in upcoming experiments.

Since X6905 showed the most potent inhibition of LPS-induced IL-6, TNF-α, CCL2, CXCL2, and CXCL10 release, we further evaluated its effects on mRNA expression of those chemokines and cytokines. Interestingly, only the expressions of IL-6, CCL2, and TNF-α were significantly reduced by pretreatment with X6905, while the expressions of CXCL2 and CXCL10 remained unaffected. In contrast to the concentration-dependent effects on the cytokine and chemokine release, 20 µM of X6905 showed a weaker inhibition of IL-6 and CCL2 release. A possible explanation for the observed differences between effects on release and expression might be the different time points of assessment. The effects of X6905 on mRNA expression were measured after 4 h of LPS stimulation, while effects on cytokine and chemokine release were measured after 24 h. Therefore, differences might be explained due to time dynamic effects. However, as we have shown previously for COX-2, the expression and synthesis differed over time, and synthesis might be modulated on post-transcriptional levels rather than by expression only [6].

When comparing the tested 3-pyridinylmethylcoumarins, X6905 exhibited the highest biological activity, followed by X7732. Interestingly, X7625 showed a tendency for contrary effects especially at lower concentrations, as observed for IL-6 and CXCL10. Due to the high electronegativity of nitrogen, the position of the N-atom in the pyridine ring may influence the affinity to receptors. This might be an explanation for the different biological effects observed between X6905, X7732, and X7625 in the current study. It seems that the pyridin-4-yl and pyridin-2-yl derivative enhance biological activity, as X6905 (pyridin-4-yl) and X7732 (pyridin-2-yl) showed higher anti-inflammatory potency. In contrast to GPR55 agonists, the antagonists at the GPR55 mostly contain a heteroaromatic or aromatic moiety attached via a methylene linker to the 3-position of the coumarin scaffold, possibly preventing receptor toggle switches. Also, the most electronegative region of commercially available GPR55 antagonists is located at the end of the molecule’s central portion, fitting vertically in GPR55’s binding pocket [38]. Regarding the coumarin scaffold, the most electronegative portion is located at the molecule’s ester function. However, the pyridine ring may be the part of the molecule protruding from the GPR55 binding pocket, stabilizing the receptor’s “off” state [38]. Further modifications to other parts of the coumarin derivatives might be even more relevant. The pyridin-3-yl derivative (X7625) might not be as effective in preventing toggle switches as the pyridin-4-yl or the pyridine-2-yl derivative. Future studies with other coumarin derivatives might help us understand the influence of chemical alterations in respect to GPR55-dependent effects as well as for coumarins acting on multiple molecular targets, referred to as “polypharmacy”. Furthermore, experimental designs to measure the affinity to GPR55 and possible other molecular targets should be pursued in future research.

Regarding the structure of the isomeric 3-pyridinylmethylcoumarins X6905, X7732, and X7625, the substitution in the C-3, C-5, and C-7 positions of the coumarin scaffolds suggests an antagonistic activity of the three compounds on the GPR55 receptor as well as an agonistic activity at CB2 due to the large lipophilic residue on C-7 [3]. Especially long and bulky alkyl moieties have been shown to increase GPR55 affinity and introducing CB agonism, possibly changing the antagonistic mechanism from orthosteric to allosteric at GPR55 [3,39]. Therefore, the 1-butylcyclohexyl residue at C-7 of X6905, X7732, and X7625 may lead to dual target molecules. For the 3-pyridinylmethylcoumarins used in this study, CB-1 and CB-2 affinities have been shown, most likely with an agonistic activity [39]. As we have shown previously, coumarin derivatives with different substitution patterns exhibited antioxidative effects, which were abolished after GPR55 knockout [5]. We further demonstrated that these compounds activate GPR55 [6], which is likely associated with their observed anti-inflammatory effects. In contrast to the coumarin derivatives evaluated in the current study, those compounds were substituted with an isopropyl group on C-8 and had no chemical residues at C-7, so no CB receptor affinity was expected [3]. However, further experiments are necessary to prove the GPR55-dependency and the significance of the expected CB-affinity on the observed anti-inflammatory effects of X6905, X7732, and X7625. Additionally, it is essential to rule out other molecular targets and potential receptor-independent effects.

Approaches to knock down the GPR55 in the BV2 microglial cells might help assign the observed effects to molecular targets, such as receptors or direct intracellular mechanisms. The anti-inflammatory effects might be mediated via the Erk-pathway, which is activated by GPR55. Lysophosphatidylinositol (LPI), discussed as endogenous GPR55 agonist, promoted phosphorylation of Erk1/2 in GPR55-HEK293 cells [40]. However, the downstream signaling of GPR55 might differ depending on the specific ligand [6,40]. Therefore, the understanding of involved pathways is crucial for the drug design and usage of functional selectivity of receptors and its associated signaling.

LPS is known to activate toll-like receptor (TLR) 4, resulting in a phosphorylation of the MAPK-pathway, leading to phosphorylation of Erk as downstream kinase and induction of pro-inflammatory gene expression via c-Fos [41]. Activation of the MAPK-Erk pathway results in a pro-inflammatory response, such as IL-6 release [42]. As a possible intracellular mechanism of the observed anti-inflammatory effects of X6905, a reduced phosphorylation of Erk1 and Erk2 was shown. However, the activation of upstream kinases of the MAPK pathway was not significantly modified by X6905 (Supplementary Materials). X7732 showed a slight trend to reduce Erk1/2 phosphorylation while X7625 did not modulate Erk phosphorylation. This is in line with the smaller biological potency of X7732 and noticeably different effects of X7625. The MAPK-Erk pathway is further activated by TNF-α, resulting in the inhibition of IL-6 release [42]. Therefore, the direct effects of X6905 on the MAPK pathway might add up with the inhibitory effects of X6905 on TNF-α release, which was not shown for X7732 and X7625. Erk1 and Erk2 are discussed as redundant isoforms, since the silencing of one gene leads to an overexpression of the remaining form. However, some studies report isoform-specific phenotypes [43]. Erk2 is expressed at higher levels in most tissues, and no isoform-specific signaling is described. Rather, Erk1 and Erk2 compete with each other for phosphorylation by MEK1/2 and share most amino acids of the enzymes’ region that interacts with the substrates [43]. Therefore, the different effects observed on phosphorylation of Erk1 and Erk2 might be dependent on the different expressional levels of both kinases.

In patients with depression, the addition of anti-inflammatory agents to an antidepressant drug enhanced therapeutic effects. Furthermore, the use of non-steroidal anti-inflammatory drugs (NSAIDs) and cytokine-inhibitors showed antidepressant effects when compared to placebo [32]. Ibuprofen, a well-known and established NSAID, is known to reduce inflammatory parameters, such as IL-6 and CCL2 [44]. However, in clinical practice, the long-term use of NSAIDs is especially associated with side-effects, such as gastric ulcers and consecutive bleeding. Another possible involved pathway is the prostaglandin E_2_ (PGE_2_) pathway via COX-2, since specific COX-2 inhibitors show antidepressant effects as adjunctive to antidepressive treatment [32,45]. However, these COX-2 inhibitors, such as celecoxib, are associated with increased cardiovascular risk and gastric bleeding as well. Via the PGE-receptor 3 (EP3), the COX-2/PGE_2_ pathway is coupled to the modulation of chemokine release, such as CCL-2 [46], and other coumarin derivatives modulate COX-2 and PGE_2_ synthesis [6,7]. Therefore, the effects of the 3-pyridinylmethylcoumarins on the COX-2/PGE_2_ pathway should be investigated further. IL-6 antibodies, such as tocilizumab, and TNF-α antibodies, such as infliximab, are already used in different diseases linked to inflammatory processes. However, studies regarding anti-depressant effects have not shown robust antidepressive effects so far [47,48], even though some studies suggest a benefit regarding depressive symptoms [49]. Those antibodies, however, are quite expensive and patients receiving those antibodies experience a higher risk of infections. Coumarin derivatives, such as warfarin, are established in the clinical use for decades as anti-coagulative drug. Beside bleeding as major complication during the warfarin treatment, the three tested 3-pyridinylmethylcoumarins might be possibly associated with less side-effects substantiating further clinical studies regarding their anti-inflammatory effects. Furthermore, other already described possible effects of the coumarin-scaffold, such as anti-microbial or anti-proliferative characteristics [2], should be evaluated in cell cultures and more complex organoid models, such as organotypic hippocampal slice cultures (OHSC), before evaluating the effects and possible side-effects in healthy and disease in vivo models.

The neuroinflammation hypothesis assumes that inflammatory mechanisms might be involved in the genesis and progression of neuropsychiatric disorders [33]. However, clinical effects of pharmacological or psychotherapeutic interventions on cytokine or chemokine levels are still on a correlative level [26,32,50,51], failing to show a causal association of central nervous inflammation and neuropsychiatric disorders. Based on the inflammatory hypothesis of depression and neuropsychiatric disorders and previous works with coumarin derivatives, the coumarin scaffold is a promising structure for the development of anti-inflammatory drugs. However, future clinical studies focusing on the inflammatory hypothesis and the potential of anti-inflammatory drugs in neuropsychiatric disorders are necessary to draw further conclusions about the potential use of the coumarin scaffold in therapeutic regimes in those disorders. Those studies should include assessment of additional effects and side-effects, since the coumarin scaffold is known for its complex pharmacological activity and multiple molecular targets [52,53]. 

## 4. Materials and Methods

### 4.1. Novel Coumarin-Derivatives X6905, X7732, and X7625

X6905, X7732, and X7625 were synthesized as previously published [39] and provided by the Karlsruhe Institute of Technology (KIT) as a 10 mM stock solution in DMSO (PAN-Biotech™ GmbH, Aidenbach, Germany). The stock solutions were stored and shipped at −20 °C.

### 4.2. BV2 Microglial Cell Culture

BV2 microglial cells were kindly provided by Prof. Langmann (Department of Ophthalmology, University of Cologne, Cologne, Germany). BV2 cells were cultured in 1x RMPI-Medium 1640 with 10% fetal calf serum (FCS) and 1% Penicillin/Streptomycin (all from Thermo Fisher Scientific Inc., Waltham, MA, USA), and kept at 37 °C and 5% CO_2_. If necessary, the medium was replaced or the cells were passaged.

For treatment, the cells’ medium was removed, rinsed with dPBS (Sigma-Aldrich Corporation, St. Louis, MO, USA), and the cells were detached, adding 0.05% Trypsin-EDTA (Thermo Fisher Scientific Inc., Waltham, MA, USA) for a few minutes. Afterwards, the cells were re-seeded in 6-, 12-, 24- or 96-well plates at different concentrations (3 × 10^5^, 2 × 10^5^, 1 × 10^5^ or 3 × 10^4^ cells/well). After cell adherence around 24 h later, the medium was replaced, and the cells were used for stimulation. Pro-inflammatory stimulation was achieved by addition of 10 ng/mL LPS from Escherichia coli O111 B:4 (from Sigma-Aldrich, Taufkirchen, Germany).

### 4.3. Cell Viability ATP Assay

An ATP assay (CellTiter-Glo^®^ 2.0 Assay from Promega Corporation, Fitchburg, WI, USA) was used to assess the cytotoxic effects of the coumarin derivatives. It works by quantifying ATP through an ATP-dependent enzymatic reaction, which emits light. To this end, the cells were plated with approximately 3 × 10^4^ cells/well in a 96-well plate. After adherence of cells, the medium was replaced before simulation. After incubation of the compounds, DMSO, and ethanol for 30 min, LPS was added for 24 h. Afterwards, the plate was incubated for 30 min at room temperature and the ATP assay reagent was added according to the manufacturer’s protocol. The plate was shaken for 2 min and incubated for another 10 min at room temperature before luminescence was detected with a Modulus™ Microplate Multimode Reader (from Promega Corporation, Fitchburg, WI, USA). The control group was set to 100% viability of BV2 cells and used as a reference for the following one-way ANOVA statistical analysis (*). Additionally, 0.2% DMSO was also used as a reference for a second one-way ANOVA (#).

### 4.4. Cell Treatment and Determination of Cytokine Release with ELISA

The cells were plated with approximately 10^5^ cells/well onto a 24-well plate. After adherence, the medium was replaced and the cells were ready for stimulation with the compounds dissolved in DMSO. To exclude the potential effects of DMSO, an equal amount of DMSO was added to untreated cells and the LPS-stimulated group, which was repeated for all following cell stimulations. Thirty minutes later, LPS was added to the appropriate wells. After 24 h of incubation, supernatants were collected and centrifuged at 11,000 rpm for 2 min to discard pellets. The samples were stored at −80 °C.

The concentrations of cytokines and chemokines in the supernatants (IL-6, CCL2, CXCL2, CXCL10, and TNF-α) were assessed by performing ELISA according to the manufacturer’s protocol (Bio-Techne GmbH, Wiesbaden, Germany).

Briefly, 96-well plates were coated with the respective capture antibody and incubated overnight. The next day, the plates were washed and blocked. After 1 h of incubation and another washing step, the samples and standards were added for 2 h. The respective detection antibody was added after another washing step. After 2 h, Streptavidin-HRP was added, and after application of 1 × TMB (Thermo Fisher Scientific Inc., Waltham, MA, USA), the plate was read at 450 nm using a MRX^e^ microplate absorbance reader (Dynex^®^ Technologies, Chantilly, VA, USA). The cytokine and chemokine release in the LPS group was set to 100% and used as reference for one-way ANOVA statistical analysis.

### 4.5. RNA Isolation and Real-Time PCR

Quantitative PCR using SYBR Green as dye was performed to investigate effects on cytokine and chemokine expression. The BV2 cells were seeded on a 6-well plate with approximately 3 × 10^5^ cells/well. The medium was replaced after cell adherence. One hour later, the cells were stimulated with X6905 and DMSO for 30 min before LPS was applied for an additional four hours. The sample collection and the RNA isolation were achieved with the Universal RNA Purification Kit (Roboklon, Berlin, Germany), the protocol was followed accordingly. After isolation, the RNA samples were solved in ddH_2_O, and the concentration was measured using NanoDrop (ddH_2_O and NanoDrop both from Thermo Fisher Scientific Inc., Waltham, MA, USA).

Subsequently, cDNA was synthesized from 500 ng purified RNA applying random primers (Biomers.net GmbH, Ulm, Germany) to the RNA sample and placed into the Thermocycler (Analytik Jena GmbH+Co. KG, Jena, Germany). After annealing, dNTPs (Sigma-Aldrich Corporation, St. Louis, MO, USA), reverse transcriptase, 5X RT-Buffer, and ribonuclease inhibitor (all from Promega Corporation, Fitchburg, WI, USA) were added and placed into the Thermocycler again for cDNA-synthesis.

For qPCR, the samples were processed on 384-well plates and incubated with SYBR green (Thermo Fisher Scientific Inc., Waltham, MA, USA) and the corresponding primers for each gene. The sequences of primers (all from Biomers.net GmbH, Ulm, Germany) were as follows. IL-6: Forward (F): 5′-AGTTGCCTTCTTGGGACTGA-3′/Reverse (R): 5′-TTCTGCAAGTGCATCATCGT-3′, CCL2: F: 5′-TGATCCCAATGAGTAGGCTGG-3′/R: 5′-ACCTCTCTCTTGAGCTTGGTG-3′, CXCL2: F: 5′-CCCTCAACGGAAGAACCAAAG-3′/R: 5′-GAGGCACATCAGGTACGATCCA-3′, CXCL10: F: 5′-CAGTGGATGGCTAGTCCTAATTG-3′/R: 5′-ACTCAGACCAGCCCTTAAAGAAT-3′, TNF-α: F: 5′-CCCACGTCGTAGCAAACCACCA-3′/R: 5′-CCATTGGCCAGGAGGGCGTTG-3′ and GAPDH: F: 5′-TGGGAAGCTGGTCATCAAC-3′/R: 5′-GCATCACCCCATTTGATGTT-3′.

The plate was set up with two technical replicates for each sample; afterwards, the mean of both values was used. To exclude self-replication of primers, an NTC containing only ddH_2_O and forward and reverse primers was also added. Consecutively expressed GAPDH was used as the reference for each sample. The qPCR was performed on the CFX384 Real Time Detection System (Bio-Rad-Laboratories, Inc., Hercules, CA, USA), and the LPS group was set to 100% of cytokine/GAPDH and used as reference for one-way ANOVA statistical analysis.

### 4.6. Electrophoresis and Immunoblotting

Western blot was used to study the effects of the compounds on intracellular signaling. The BV2 cells were plated at approximately 2 × 10^5^ cells/well onto a 12-well cell culture plate. After adherence, the medium was replaced, and the cells were stimulated after 1 h. The compounds and DMSO were added for 30 min before stimulation with LPS for another 30 min. The medium was removed, and all wells were rinsed with dPBS twice. To prevent protein-lysis, all steps were performed on ice. A total of 100 µL of lysis buffer (LB)++ (containing 42 mM Tris-HCl, 1.3% sodium dodecyl sulfate, 6.5% glycerin, 100 µM sodium orthovanadate, and 2% phosphatase/0.2% protease inhibitors) was added to each well, and cell lysis was achieved through scratching the cells with an upside-down pipette tip for 1 min. After the sample collection, the lysate was centrifuged and heated at 90 °C for 5 min.

To determine the protein concentration of each sample, a BCA Protein Assay (Thermo Fisher Scientific Inc., Waltham, MA, USA) was performed after the sample collection. The protocol was followed accordingly, and the samples were read using a microplate reader at 570 nm. DTT (Sigma-Aldrich Corporation, St. Louis, MO, USA) was added to each sample before dilution in a loading buffer to a concentration of 0.5 µg/µL. For SDS-PAGE, 7.5 µg of each sample was run on a 10% acrylamide gel. Afterwards, the proteins were blotted onto a 3 mm transfer membrane (Sigma-Aldrich Corporation, St. Louis, MO, USA), blocked for 1 h with Roti-Block (Carl Roth GmbH+Co. KG, Karlsruhe, Germany) and washed with TBS-T for 10 min before adding the primary antibody and incubating at 4 °C overnight.

The following primary antibodies were used: mouse anti-vinculin (Cat. No.: V9264- 25UL, diluted 1:20,000, Sigma-Aldrich Corporation, St. Louis, MO, USA); rabbit anti-phospho-p44/42 MAPK (Cat. No.: 45899, diluted 1:1000, Cell Signaling Technology, Cambridge, UK); Phospho-SAPK/JNK (Cat. No.: 9251S, diluted 1:1000, Cell Signaling Technology, Cambridge, UK); Phospho-PKC (pan) (Cat. No.: 9371T, diluted 1:1000, Cell Signaling Technology, Cambridge, UK); Phospho-p38 MAPK (Cat. No.: 4511S, diluted 1:1000, Cell Signaling Technology, Cambridge, UK), and IkBα antibody (Cat. No.: 9242S, diluted 1:1000, Cell Signaling Technology, Cambridge, UK). After washing three times with TBS-T for 10 min, the respective secondary antibody (anti-mouse secondary antibody: Cat. No.: 401253-2ML, diluted 1:10,000, Merck KGaA, Darmstadt, Germany, or anti-rabbit secondary antibody: Cat. No.: 5450-0010, diluted 1:10,000, LGC Ltd., Teddington, UK) was added and incubated for at least 1 h. After another three washing steps with TBS-T, quantification of the protein-bound antibodies through the application of WesternBright chemiluminescence substrate (Biozym Scientific GmbH, Hessisch Oldendorf, Germany) was performed using the ChemiDoc™ MP Imaging System (Bio-Rad-Laboratories, Inc., Hercules, CA, USA; Image Lab Touch software version 2.2.0.08). ImageJ 1.54g (National Institute of Health, Bethesda, MD, USA) was used for the densiometric analysis of the chemiluminescence bands. Vinculin, an underlying constitutive expression, was used for the normalization of the quantity of each sample, and the values are shown as protein/vinculin. The LPS group was set to 100% of protein/vinculin and used as reference for one-way ANOVA statistical analysis.

### 4.7. Statistical Analysis

The raw values were first converted into percentages of the corresponding reference group (untreated cells for ATP-assay or LPS for ELISA, qPCR, and Western blot). All data are presented as the mean of each value of the group ± SDs of three or more biological replicates. The statistical analysis was performed using one-way ANOVA with GraphPad Prism 10.2.3 (Graphpad Software, Inc., La Jolla, CA, USA). The level of significance is shown as * *p* < 0.05, ** *p* < 0.01, *** *p* < 0.001, **** *p* < 0.0001 and indicated above the respective bar. 

## 5. Conclusions

Anti-inflammatory effects have been described for C3-, C5-, and C7-substituted coumarins and are most likely dependent on an inverse agonistic activity at GPR55. In previous studies, we already showed the relevance of different chemical residues on the anti-inflammatory potency. However, in this study, we proved that the position of nitrogen in the pyridine ring is crucial for the biological effects of the isomeric coumarin derivatives. These findings might offer new insights for the development of a new class of anti-inflammatory pharmaceutics that might open new therapeutic options in the treatment of psychiatric disorders.

## Data Availability

All data on the synthesis and analysis of the investigated compounds, X6905, X7732, and X7625, are available via the Chemotion repository (https://www.chemotion-repository.net/, accessed on 18 January 2025) and can be accessed via the following collection DOI: https://dx.doi.org/10.14272/collection/CSR_2024-10-07 [54].

## Supplementary Materials

The following are available online at https://www.mdpi.com/article/10.3390/molecules30112452/s1, Figure S1. Effects of X6905 (light blue bars) on phospho p38 MAPK, Vinculin was used as reference for equal gel loading. Cells were stimulated with X6905 for 30 min, before LPS was added for another 30 min. Values were normalized to LPS and are shown as the mean of 3 individual Western Blot measurements with ± SDs. One-way-ANOVA with Dunett’s post hoc test was performed using the LPS-group (black bar) as reference; Figure S2. Effects of X6905 (light blue bars) on phospho PKC beta, Vinculin was used as reference for equal gel loading. Cells were stimulated with X6905 for 30 min, before LPS was added for another 30 min. Values were normalized to LPS and are shown as the mean of 3 individual Western Blot measurements with ± SDs. One-way-ANOVA with Dunett’s post hoc test was performed using the LPS-group (black bar) as reference; Figure S3. Effects of X6905 (light blue bars) on IkBa, Vinculin was used as reference for equal gel loading. Cells were stimulated with X6905 for 30 min, before LPS was added for another 30 min. Values were normalized to LPS and are shown as the mean of 3 individual Western Blot measurements with ± SDs. One-way-ANOVA with Dunett’s post hoc test was performed using the LPS-group (black bar) as reference.
