# Supplementary material for: Isomeric 3-Pyridinylmethylcoumarins Differ in Erk1/2-Inhibition and Modulation of BV2 Microglia-Mediated Neuroinflammation
Source: Molecules. 2025 Jun 3;30(11):2452. doi: 10.3390/molecules30112452 (PMC12156390; doi:10.3390/molecules30112452)
Supplement: Supplementary file 1 [file molecules-30-02452-s001.zip › molecules-3648074-supplementary.pdf]

Supplementary Material

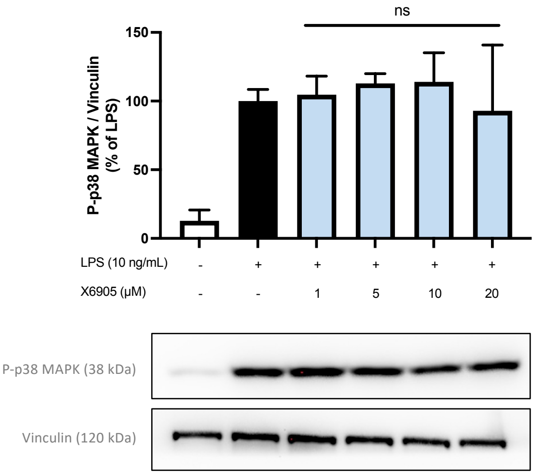

Figure S1. Effects of X6905 (light blue bars) on phospho p38 MAPK, Vinculin was used as reference for equal gel loading. Cells were stimulated with X6905 for 30 minutes, before LPS was added for another 30 minutes. Values were normalized to LPS and are shown as the mean of 3 individual Western Blot measurements with ± SDs. One-way-ANOVA with Dunett's post hoc test was performed using the LPS-group (black bar) as reference.

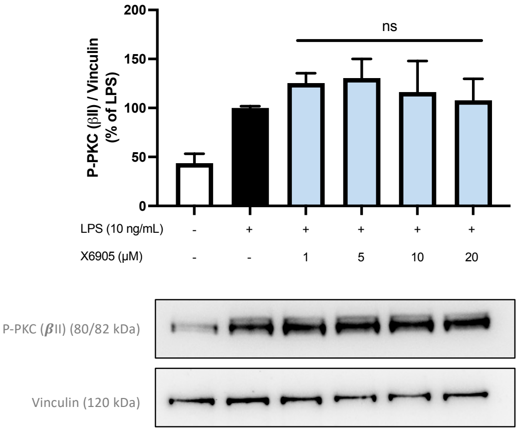

Figure S2. Effects of X6905 (light blue bars) on phospho PKC beta, Vinculin was used as reference for equal gel loading. Cells were stimulated with X6905 for 30 minutes, before LPS was added for another 30 minutes. Values were normalized to LPS and are shown as the mean of 3 individual Western Blot measurements with ± SDs. One-way-ANOVA with Dunett's post hoc test was performed using the LPS-group (black bar) as reference.

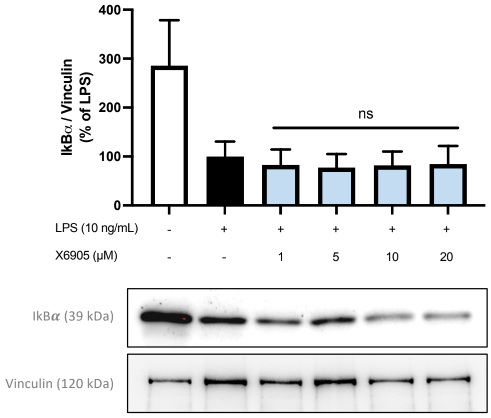

Figure S3. Effects of X6905 (light blue bars) on IkBα, Vinculin was used as reference for equal gel loading. Cells were stimulated with X6905 for 30 minutes, before LPS was added for another 30 minutes. Values were normalized to LPS and are shown as the mean of 3 individual Western Blot measurements with ± SDs. One-way-ANOVA with Dunett's post hoc test was performed using the LPS-group (black bar) as reference.
